# Creep to inertia dominated stick-slip behavior in sliding friction modulated by tilted non-uniform loading

**DOI:** 10.1038/srep33730

**Published:** 2016-09-19

**Authors:** Pengyi Tian, Dashuai Tao, Wei Yin, Xiangjun Zhang, Yonggang Meng, Yu Tian

**Affiliations:** 1 State Key Laboratory of Tribology, Tsinghua University, Beijing 100084, China

## Abstract

Comprehension of stick-slip motion is very important for understanding tribological principles. The transition from creep-dominated to inertia-dominated stick-slip as the increase of sliding velocity has been described by researchers. However, the associated micro-contact behavior during this transition has not been fully disclosed yet. In this study, we investigated the stick-slip behaviors of two polymethyl methacrylate blocks actively modulated from the creep-dominated to inertia-dominated dynamics through a non-uniform loading along the interface by slightly tilting the angle of the two blocks. Increasing the tilt angle increases the critical transition velocity from creep-dominated to inertia-dominated stick-slip behaviors. Results from finite element simulation disclosed that a positive tilt angle led to a higher normal stress and a higher temperature on blocks at the opposite side of the crack initiating edge, which enhanced the creep of asperities during sliding friction. Acoustic emission (AE) during the stick-slip has also been measured, which is closely related to the different rupture modes regulated by the distribution of the ratio of shear to normal stress along the sliding interface. This study provided a more comprehensive understanding of the effect of tilted non-uniform loading on the local stress ratio, the local temperature, and the stick-slip behaviors.

The onset of friction is a basic and important problem in the understanding of the principle of tribology. As well known, the frictional force in a sliding friction usually depends on both the real contact area and the shear strength of each micro-contact^1^,^2^. However, many previous sliding friction tests were conducted through macroscopic measurement, thus neglected the micro-contact behaviors. The interaction between two sliding surfaces has mostly been evaluated with empirical laws^3^,^4^,^5^. Therefore, the study on micro-contact dynamics and their frictional strength evolution has been paid more attention recently^6^,^7^,^8^,^9^. The interfacial shear strength has been found to be inherently dependent on the competition between the process of detachment and re-attachment of the micro-contacts on the interface^10^,^11^,^12^,^13^, and the contact area rejuvenation^1^,^14^,^15^. According to the different time scales of these processes, the dynamics of the interface changed with the sliding velocity^16^,^17^,^18^. At a slow velocity, the dynamics of the micro-contacts is creep-dominated, if the stick time long enough that the aging of asperities plays a leading role^14^,^19^,^20^,^21^. When the sliding velocity is high enough, the asperities have no time to creep before slip occurs, the dynamics of stick-slip turns to be inertia-dominated. However, the micro-contact behaviors in the transition from creep- dominated to inertia-dominated regime are still lack of comprehensive experimental verification.

Oded Ben-David *et al*.^15^ experimentally studied the evolution of frictional strength in extremely short to long time scales through continuous measurement of the evolution of the real contact area. They found that different rupture modes (supershear, sub-Rayleigh and slow rupture) were related to the ratio of shear stress to normal stress^22^. In this study, the creep-dominated to inertia-dominated dynamics during stick-slip and the interface rupture of two polymethyl methacrylate (PMMA) blocks were experimentally investigated. The sliding friction behavior of the sliding interface was modulated by slightly tilt the angle between the two sliding blocks to realize a non-uniform loading and a change of distribution of the ratio of local shear stress to normal stress.

Considering that the detachment and reattachment of the asperities during sliding friction usually occurs in a very short time and the energy of deformation and rupture would release in the form of elastic waves during stick-slip, acoustic emission (AE) has been widely used in the study of stick-slip especially in the crack of rocks concerning earthquake^23^,^24^,^25^,^26^,^27^. The previous researches mostly concerned on the time-domain features of AE during stick-slip^28^ and its relationship with the intensity of macro stick-slip events^29^. Therefore, in this study, we also detected the AE signals during stick-slip processes to help the understanding of their mechanisms.

## Results

### Stick-slip behaviors modulated by the tilt angle

**Figure 1:**
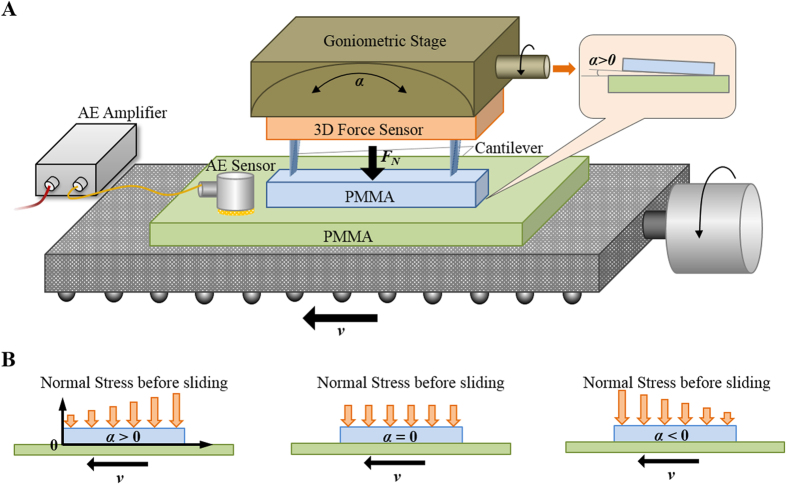
Schematic of the experimental setup. (**A**) Stick-slip experimental system. (**B**) Tilt angle definition.

**Figure 2:**
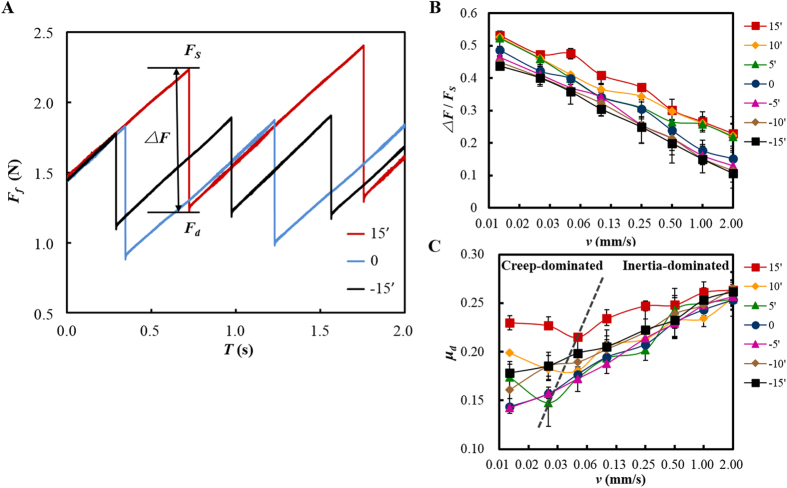
Stick-slip behaviors at different tilt angles. (**A**) Typical curves of the frictional force *F*_*f*_ during stick-slip under different tilt angles (*F*_*N*_ = 6 N, *v* = 0.025 mm/s). (**B**) Statistical results of the stick-slip intensity *ΔF*/*F*_*S*_. (**C**) Statistical results of the dynamic friction coefficient *μ*_*d*_. The dashed line connected the turning point at different positive tilt angles showing the trend of *v*_*t*_ which indicates the two dynamic regimes. (Each data point was the average of 5 to 15 different tests at the same experimental condition with the error bars showing the fluctuation of each test.)

Stick-slip experiments were conducted with two PMMA blocks using the test apparatus shown in Fig. 1. Considering the rupture dynamics of micro-contacts was controllable by external loading condition^30^, we changed the load distribution along the sliding interface by slightly tilting the upper PMMA block with a small tilt angle *α* under a certain sliding velocity *v*. The definition of the tilt angle and the corresponding stress distribution was sketched in Fig. 1B. When the lower block was pulled from a higher normal stress side to a lower normal stress side, the tilt angle was defined to be positive (the tilt direction was sketched in the inset of Fig. 1A). The inverse condition was defined to be negative. Details of the experiments were given in Methods. Typical curves of the frictional force *F*_*f*_ during stick-slip at different tilt angles were shown in Fig. 2A. Results showed that the stick-slip motion was enhanced as the increase of tilt angle. *ΔF* is the difference between the maximum static friction force *F*_*S*_ and the minimum tangential force *F*_*d*_ after slip. As the normal load would significantly affect the value of *ΔF*, here we use the normalized parameter*ΔF*/*F*_*S*_ to characterize the stick-slip intensity which excludes the effect of the normal load. From the statistical results shown in Fig. 2B*ΔF*/*F*_*S*_ slightly increases with the increasing tilt angle, indicating the enhancement of stick-slip motion, and decreases almost linearly with the increasing sliding velocity. Based on previous researches, the stick-slip motion would experience creep-dominated to inertia-dominated dynamic regimes with the increasing sliding velocity^16^, which is characterized by the different trends of the dynamic friction coefficient *μ*_*d*_ (defined as the ratio of *F*_*d*_ to the normal force *F*_*N*_) with the sliding velocity. At a low sliding velocity, creep and aging effect of the contact asperities in the stick stage was obviously, during which *μ*_*d*_ decreases with the increasing *v*. When the specimen is pulled at a velocity which is high enough that gives no time for the asperities to creep before slip occurs, the sticks-slip motion turns to be inertia-dominated, during which *μ*_*d*_ increases with *v*^16^,^18^. From our results in Fig. 2C, at positive tilt angles, *μ*_*d*_ first decreases with *v*, indicating the creep-dominated stick-slip, and then turns to increase with *v* which means the stick-slip dynamics turns into inertia-dominated. As shown by the dashed line, the turning velocity *v*_*t*_ increases slightly with the tilt angle. And for 0 and negative *α*, the creep-dominated regime does not show in our experimental velocity range. From the results in Fig. 2, the non-uniform loading configuration by a tilt angle could not only affect the intensity but also the dynamics of stick-slip.

### Stick-slip dynamics reflected by AE

**Figure 3:**
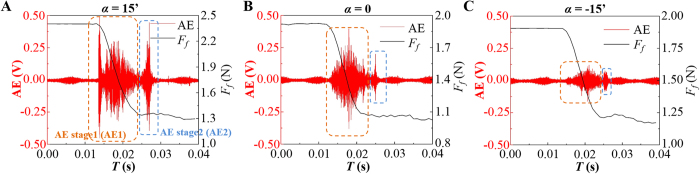
Typical AE waves during one slip event at three different tilt angles. (**A–C**) *α* = 15′, 0 and −15′, respectively. Two stages of the AE signal: AE1, excited during the main slip; AE2, excited during the tremor after slip.

**Figure 4:**
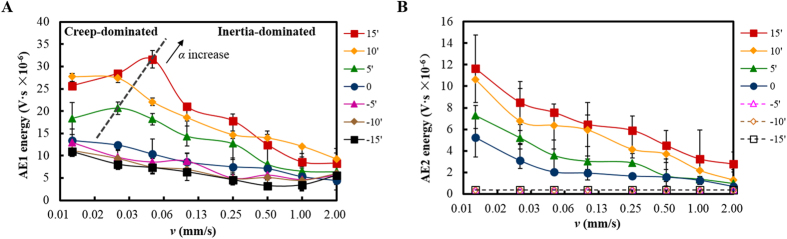
Statistical results of the AE energy at different sliding velocities and tilt angles (*F*_*N*_ = 6 N). (**A**) Energy of AE signal in stage 1. The dashed line connected the turning point at different positive tilt angles indicating the two dynamic regimes). (**B**) Energy of AE signal in stage 2. (Each data point was the average of 5 to 15 different tests at the same experimental condition with the error bars showing the fluctuation of each test.)

**Figure 5:**
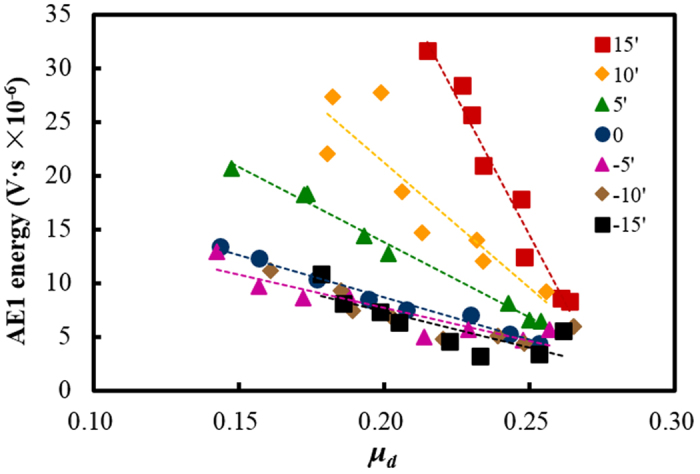
Energy of the AE signals in stage 1 versus the dynamic friction coefficients at different tilt angles. The dashed lines showed the linear fitting of the data.

The acoustic emission could represent the elastic energy emission during the detachment and re-attachment of asperities. Typical AE waves during stick-slip process at different tilt angles were shown in Fig. 3. A burst of AE wave was excited at the beginning of slip, followed by a series of small waves. The AE signal during the slip process was defined as the first AE stage (AE1). During the tremor right after the slip, another AE wave was excited and was defined as the second AE stage (AE2). As shown in Fig. 3, the AE signal in both stages decreased with the decrease of the tilt angle. The first burst-type AE could not be observed for zero and negative tilt angles. Figure 4 shows the detected AE energy (envelope area of the AE wave) of the two stages corresponding to the stick-slip motions in Fig. 2B. As shown in Fig. 4A, AE1 energy exhibited an opposite trend to *μ*_*d*_. As shown in Fig. 5, the AE1 energy decreased approximately linearly with *μ*_*d*_, and the slope increased with the tilt angle. This relationship between AE1 energy and *μ*_*d*_ indicates that AE in stage 1 may be closely related to the stick-slip dynamics. While, the energy of AE2 decreased monotonically with the sliding velocity (Fig. 4B) in accordance with *ΔF*/*F*_*S*_ (for negative tilt angles, AE2 was too weak to be distinguished from the background noise). This indicates that AE in stage 2 is only determined by the stick-slip intensity.

### Simulation of tilt angle modulation on load distribution

**Figure 6:**
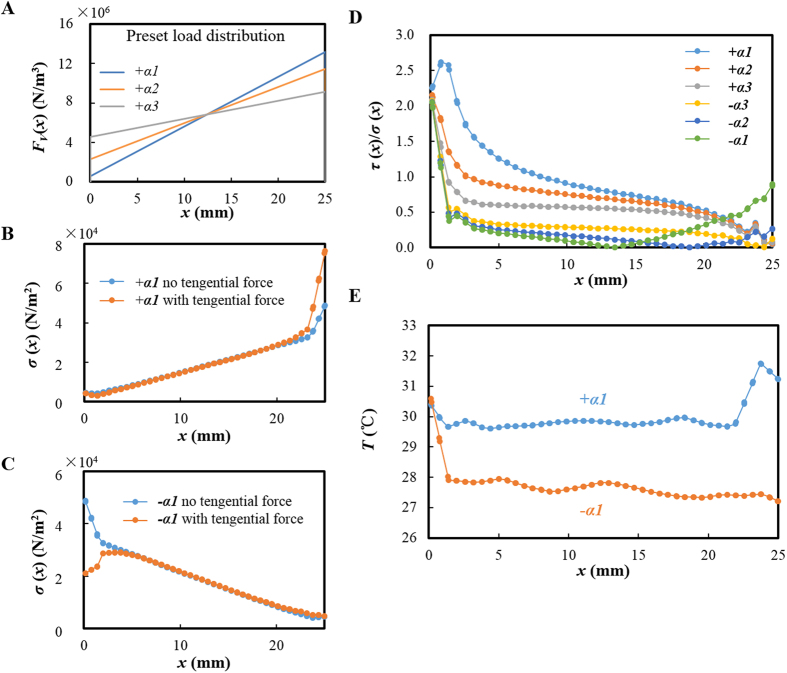
Finite element analysis of the effect of tilt angle. (**A**) Original normal load per unit volume *F*_*V*_ (body load) applied on the upper block along the interface at three tilt angles. (**B**) FE simulation result of the interface normal stress (per unit area) distribution along the block with or without tangential force at positive tilt angle. (**C**) FE simulation result of the interface normal stress distribution along the block with or without tangential force at negative tilt angle. (**D**) Ratio of shear stress to normal stress along the interface at different tilt angles. (**E**) Surface temperature distribution along the interface for both positive and negative tilt angles.

As the tilt angle would significantly affect the stress distribution along the sliding interface^30^, a finite element (FE) analysis was carried out in this study by using Comsol 5.1 to analyze the tilt angle modulated non-uniform loading effect. The non-uniform loading applied on the upper block was simulated by a linear increasing normal force along the interface as shown in Fig. 6A (*α1* > *α2* > *α3* represents the increasing non-uniformity of the original loading). More details of the FE model was supplied in Methods. Due to the elasticity of the material and the boundary effect, the calculated initial normal stress profile along the interface showed little difference with the preset values, especially at the two edges as shown in Fig. 6B,C. After applying a tangential friction force, the normal stress distribution was changed by the *F*_*S*_ induced additional torque, consistent with previous research^30^. At a positive tilt angle, the normal stress was enhanced at the leading edge (*x* = 25 mm), while was weakened at the trailing edge (*x* = 0 mm), resulting in a more asymmetric load distribution. At a negative tilt angle, the *F*_*S*_ induced torque could decrease the normal stress at the trailing edge and weaken the asymmetry of load distribution.

In previous studies, the dynamics of slip initiation and the interface rupture modes were found to be determined by the local ratio of shear stress (*τ*(x)) to normal stress (*σ*(x))^22^,^30^. The stress ratio (*τ*(x)/*σ*(x)) in this simulation was shown in Fig. 6D. The high ratio of tangential to normal stress appears at the trailing edge for both positive and negative tilt angles, where the rupture front would nucleate and then propagate along the interface^22^,^30^. When *α* > 0, supershear ruptures (*τ*(x)/*σ*(x) > 1) firstly started near the trailing edge, and then turned into sub-Rayleigh mode (0.5 < *τ*(x)/*σ*(x)<1) during propagation. When *α *< 0, *τ*(x)/*σ*(x) decreased sharply from about 2 at the trailing edge to a much lower value (mostly smaller than 0.5) elsewhere along the interface. This indicated a slow rupture dominated stick-slip behavior at negative tilt angles^22^.

By comparing the simulation results with the AE waves shown in Fig. 3, AE1 was considered to be closely related to the rupture modes: a higher velocity of the rupture front led to a stronger AE1 signal. The first burst-type of AE was considered to appear when *τ*(x)/*σ*(x) is larger than a certain critical high value, so it could only be observed for a positive tilt angle large enough. When *α *< 0, the shear stress along the interface and the rupture velocity were both low, so the released AE energy was much smaller, reflected by the weak signals in Fig. 3C. From Fig. 6D, *τ*(x)/σ(x) increased monotonously with the positive tilt angle, so the AE energy showed a significant increase with *α* (>0) in Fig. 4A. However, when *α *< 0, decreasing the tilt angle, the stress ratio would decrease from the trailing edge but increase on the other side. This led to an insignificant change of the AE energy as shown in Fig. 4A.

### Simulation of tilt angle modulated thermal behavior at the interface

For PMMA specimens, the mechanical property of asperities was found to be significantly affected by temperature^11^,^31^. Therefore, we coupled thermal analysis with the above mechanical FE simulation (see Methods for details). Taking a slip velocity of 0.5 m/s (in the same order of the experimental value) and a slip time of 0.01 s, the simulated temperature distribution along the interface was shown in Fig. 6E. The temperature at a positive tilt angle was much higher than that at a negative tilt angle, especially at the leading edge. It should be noted that since the contact surfaces were assumed to be flat in the simulation, the FE result was the mean bulk surface temperature. In real cases, because of the surface roughness, the flash temperature of the asperities would have the same trend with, but should be much larger than the values shown in Fig. 6E. The high temperature could soften the asperities and increase their mechanical deformation and adhesion, which agreed with the increased static friction coefficient *μ*_*S*_ with the normal load as shown in Fig. S1. Increasing the tilt angle, the normal stress at the leading edge would be enhanced more, resulting in a higher temperature and a higher *ΔF* and *ΔF/F*_*S*_ (Fig. 2A).

From Fig. 2B, the critical velocity *v*_*t*_ corresponding to the transition from creep-dominated to inertia-dominated stick-slip increased slightly with *α*, which could be explained from the following two aspects. On one hand, the stick process was strengthened by the temperature increase at a positive tilt angle. The increasing stick time would enhance the creep and aging of the asperities^14^,^18^. On the other hand, the positive tilt angle enhanced the normal stress at the leading edge. As discussed above, rupture started from the trailing edge, so the asperities near the leading edge would have more time to creep at a larger normal stress. While, the cases at negative tilt angles were just the opposite: the creep effect was weakened by decreasing the maximum normal stress^18^, resulting in the decrease of *v*_*t*_.

The results of *μ*_*d*_ in Fig. 2C indicated that the creep of asperities was strengthened with the decrease of sliding velocity. A lower sliding velocity gave more time for the asperities at the leading edge to creep. The deformed and softened asperities by the high temperature would absorb more elastic wave energy released from the crack on the trailing edge. Therefore, the AE1 energy decreased with the decrease of velocity in the creep-dominated regime in Fig. 4A. The creep effect of asperities could be ignored in the inertia-dominated regime. The energy intensity of the emitted elastic waves depended on the slip intensity. Therefore, the AE1 energy turned to decrease with the increase of sliding velocity, along with the change of *ΔF*/*F*_*S*_.

In summary, the stick-slip dynamics was studied from the perspective of interfacial rupture of two PMMA blocks using the acoustic emission (AE) technique. The dynamics of stick-slip motion from creep-dominated to inertia-dominated was modulated by the tilt angle between the contact surfaces through changing the normal stress distribution by the *F*_*S*_ induced torque. The various stick-slip dynamics was reflected by the AE signals. The AE signal emitted in slip process was related to the dynamics of stick-slip behaviors. The AE signal in the tremor at the end of the slip was related to the intensity of the stick-slip event. The tilt angle modulated stick-slip was affected by the non-uniform loading and the resulted non-uniform interfacial temperature distribution and their effects on the mechanical properties of materials at the sliding interface.

## Methods

### Experimental methods and procedures

Two PMMA blocks were pressed together with a normal force *F*_*N,*_ as shown in Fig. 1. The upper specimen (25 × 5 × 7 mm^3^) was fixed on the force sensor through a cantilever a stiffness of 1.038 N/mm to adjust the tangential stiffness of the system. The lower specimen (91 × 50 × 3 mm^3^) was drove by a motor with a sliding velocity *v*. The surface roughnesses of the upper and lower specimen were 1.6 nm and 2.6 μm, respectively.

An AE sensor (PICO-1.2HF, Physical Acoustics Corporation) was coupled onto the lower specimen surface with high vacuum grease. Its operating frequency range was from 250 to 1700 kHz, and the resonant frequency was 550 kHz.

The stress profile along the interface was modulated by changing the tilt angle *α* of the two blocks by the goniometric stage. Resolution of the angle adjustment was 5′. When the lower block was pulled from the high normal stress side to the low normal stress side, the tilt angle was defined as positive (Fig. 1B). The inverse direction was defined as negative.

To obtain the initial zero tilt angle, a pre-adjustment and sliding was conducted. The lower block was pulled in two opposite directions respectively under a normal load *F*_*N*_ of 4 N. The tangential force was simultaneously measured. After repeating adjustments, the position was defined as *α* = 0 when the tangential forces of the two directions was almost the same (relative error less than 5%). Then the main tests were conducted. *F*_*N*_ was set as 6 N. By adjusting the goniometric stage, the stick-slip behaviors were tested at 7 tilt angles (−15′, −10′, −5′, 0°, 5′, 10′, 15′). For each angle, the sliding velocity was 0.01, 0.025, 0.05, 0.1, 0.25, 0.5 and 1, 2 mm/s, respectively. The stroke was 8 mm. The acquisition rate was 1000 Hz for the tangential force, and 2 MHz for the AE signals. Each test was repeated 5–15 times under the same condition. All experiments were conducted at room temperature (about 28 °C) and a relative humidity of approximately 40%.

### Finite element analysis

FE analysis was conducted using Comsol 5.1. A 3D geometry model was established according to the actual size of the specimens. The tilted non-uniform loading was simulated by linear increasing original normal body load *F*_*V*_ (normal load per unit volume) along the interface of the upper block, as shown in Fig. 6A (*α1* > *α2* > *α3*, which coordinate system was shown in Fig. 1B). And an original friction force was applied on the interface of the upper specimen in two opposite directions for positive and negative tilt angles respectively (the direction of *v* shown in Fig. 1B) to simulate the sliding. Taking 0.5 as the average maximum stick friction coefficient, the value of this original friction force was set as half of normal stress at every point along the interface. The top surface of the upper specimen and the bottom surface of the lower specimen were set as constraint boundaries.

In thermal analysis, the dynamic friction coefficient during slip was set as 0.25. The interface of the two specimens was set as the boundary heat source. Assuming the frictional power all converted into heat for simple, the heat power was the product of the shear stress from the above mechanical module and the slip velocity. Surfaces exposed in the air were all set as heat radiation boundaries.

Steady-state calculation was used for the stress simulation, and transient calculation for the thermal simulation. The simulation time was set as 0.01 s with a step time of 0.001 s. The variables and expressions used in the simulation were shown in Tables S1 and S2.

## Additional Information

**How to cite this article**: Tian, P. *et al*. Creep to inertia dominated stick-slip behavior in sliding friction modulated by tilted non-uniform loading. *Sci. Rep.* **6**, 33730; doi: 10.1038/srep33730 (2016).

## Supplementary Material

Supplementary Information
