# Supplementary material for: Creep to inertia dominated stick-slip behavior in sliding friction modulated by tilted non-uniform loading
Source: Sci Rep. 2016 Sep 19;6:33730. doi: 10.1038/srep33730 (PMC5027382; doi:10.1038/srep33730)
Supplement: Supplementary Information [file srep33730-s1.doc]

**Supplementary Information**

# Creep to inertia dominated stick-slip behavior in sliding friction modulated by tilted non-uniform loading

Pengyi Tian, Dashuai Tao, Wei Yin, Xiangjun Zhang, Yonggang Meng, Yu Tian*

State Key Laboratory of Tribology, Tsinghua University, Beijing 100084, China

*Corresponding: [tianyu@mail.tsinghua.edu.cn](mailto:tianyu@mail.tsinghua.edu.cn)

| Symbol | Description | Value | |
| --- | --- | --- | --- |
|  |  | PMMA specimen | Air |
| *ρ* | Density (kg/m^3^) | 1175 | 1.1697 |
| *C* | Specific heat capacity (J/(kg·k)) | 1464 | 1100 |
| *λ* | Thermal conductivity (W/(m·k)) | 0.18 | 0.026 |
| *μ* | Dynamic viscosity (Pa·s) |  | 1.08×10^-3^ |
| *e* | Emissivity | 0.9 |  |
| *E* | Elastic Modulus (GPa) | 3.16 |  |
| *υ* | Poisson's ratio | 0.32 |  |
| *l* | Length of the interface (mm) | 25 | |
| *w* | Width of the interface (mm) | 5 | |
| *h* | Height of the upper specimen (mm) | 7 | |
| *T_air* | Ambient temperature (℃) | 26.85 | |
| *cof* | Friction coefficient | 0.5 (for static stress simulation)  0.25 (for thermal simulation in slip) | |
| *v_slip* | Slip velocity (m/s) | 0.5 | |

**Table S1.** Variables used in the simulation.

| Symbol | Description | Expression |
| --- | --- | --- |
| *F_V_*(*x*) | Normal body load distribution (N/mm^3^) | *α1*: 11*x*/(*l*^2^·*w*·*h*)+1/(2*l*·*w*·*h*)  *α2*: 8*x*/(*l*^2^·*w*·*h*)+2/(2*l*·*w*·*h*)  *α3*: 4*x*/(*l*^2^·*w*·*h*)+4/(2*l*·*w*·*h*) |
| *τ_0_*(*x*) | Shear stress distribution (N/mm^2^) | *F_V_*(*x*)×*h*×*cof* |
| *q_prod*(*x*) | Heat power distribution (W/mm^2^) | *τ*(*x*)×*v_slip*  (*τ*(*x*) is the result from the stress simulation.) |

**Table S2.** Variable expressions used in the simulation

**Figure S1.** Static friction coefficient versus the sliding velocity under different loads with 0 tilt angle.
